# Genomic and Population-Level Effects of Gene Conversion in *Caenorhabditis* Paralogs

**DOI:** 10.3390/genes1030452

**Published:** 2010-12-09

**Authors:** Vaishali Katju, Ulfar Bergthorsson

**Affiliations:** Department of Biology, University of New Mexico, Albuquerque, NM 87131, USA; E-Mail: ulfar@unm.edu

**Keywords:** *Caenorhabditis*, conversion tract, ectopic gene conversion, *ftr-1*, *fog-2*, gene duplication, interlocus gene conversion, paralogs

## Abstract

Interlocus gene conversion, the nonreciprocal exchange of genetic material between genes, is facilitated by high levels of sequence identity between DNA sequences and has the dual effect of homogenizing intergenic sequences while increasing intragenic variation. Gene conversion can have important consequences for the evolution of paralogs subsequent to gene duplication, as well as result in misinterpretations regarding their evolution. We review the current state of research on gene conversion in paralogs within *Caenorhabditis elegans* and its congeneric species, including the relative rates of gene conversion, the range of observable conversion tracts, the genomic variables that strongly influence the frequency of gene conversion and its contribution to concerted evolution of multigene families. Additionally, we discuss recent studies that examine the phenotypic and population-genetic effects of interlocus gene conversion between the sex-determination locus *fog-2* and its paralog *ftr-1* in natural and experimental populations of *C. elegans*. In light of the limitations of gene conversion detection methods that rely solely on the statistical distribution of identical nucleotides between paralogs, we suggest that analyses of gene conversion in *C. elegans* take advantage of mutation accumulation experiments and sequencing projects of related *Caenorhabditis* species.

## 1. Introduction

The expansion of gene number within organisms is facilitated by the processes of gene duplication and polyploidization. The study of gene duplicates has burgeoned within the last two decades, owing largely to the availability of whole-genome sequences that enable the identification of a genome-wide population of paralogs for determining the dominant pattern(s) of duplicate origin and the evolutionary forces responsible for their diversification and retention, as well as new methods to assess copy-number variation in natural populations. Additionally, these large data sets enable exploration of additional hypotheses that hitherto remain unanswered (e.g., which evolutionary forces drive the fixation of duplicates at the species-wide level) and test key theoretical predictions for gene duplicate evolution that were established in the pre-genomic era [1,2,3]. 

The canonical model of gene duplicate evolution as outlined by Ohno [1] postulates that gene duplicates originate bearing complete sequence and functional identity to the progenitor copy (*i.e*., ancestral gene structure and the entire repertoire of ancestral regulatory elements are preserved during the gene duplication process). Sequence divergence between the two paralogs over evolutionary time is affected by the gradual accumulation of mutations, ultimately leading to functional divergence or pseudogenization and eventual loss. However, we now know that this view of gene paralog evolution is overly simplistic. Paralogs are also capable of nonreciprocal recombination with each other via gene conversion, a form of concerted evolution wherein one donor sequence converts a homologous recipient sequence over some length of sequence, thereby enhancing sequence homogeneity between the two paralogs [4]. Hence, the evolutionary trajectories of gene paralogs is governed by the relative frequencies of these two opposing forces; sequence divergence by new mutations and the erosion of sequence heterogeneity via gene conversion [5,6]. 

The 1980s witnessed accumulating evidence for gene conversion among paralogs representing a range of functions across a variety of species such as human α- and goat ξ-globins [7,8,9], *Drosophila* heat-shock genes [10], human fetal globin genes [11], mouse immunoglobulin α-heavy chain constant regions [12], and chorion genes in *Bombyx* [13], among others. The earliest detailed insights regards the biological mechanics of ectopic gene conversion events are owing to genetic studies in microorganisms, particularly the yeast *Saccharomyces cerevisiae* [14]. Interlocus gene conversion events were studied by taking advantage of lines bearing naturally-occurring repetitive elements [15] as well as experimentally-manipulated strains bearing an insertion of an extra, synthesized gene copy [16,17,18,19] to determine the influence of a homolog’s genomic location on the frequency of gene conversion. The yeast system has been further developed to determine the relative frequencies of intralocus [20] *versus* interlocus (ectopic) gene conversion [17,21,22], the physical length of gene conversion tracts [23,24,25], the degree of bias with respect to the identity of the donor sequence during gene conversion [26], and the genome-wide rates of gene conversion events [27]. However, Li [4] has cautioned that generalizations from patterns and rates of gene conversion in yeast may have limited applicability to other eukaryotic genomes due to an extremely high rate of gene conversion in yeast and the widespread presence of paralogs as templates for gene conversion in its genome given its polyploid history [28,29]. The nematode *Caenorhabditis elegans* may represent an appropriate multicellular eukaryotic model system to study the consequences of gene conversion for the evolutionary dynamics of multigene families in a genome dominated by small-scale duplication events (in contrast to polyploidy).

## 2. Detection of Gene Conversion in *Caenorhabditis* Paralogs

**Figure 1:**
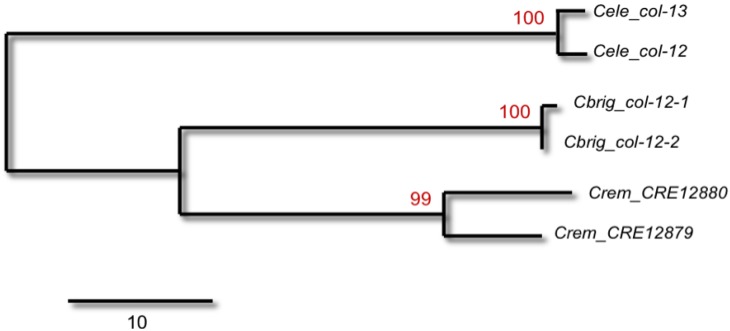
A maximum-parsimony tree of the relationship between *C. elegans* collagen genes *col-12* and *col-13* and their orthologs in *C. remanei* and *C. briggsae* using protein-coding DNA sequences downloaded from Wormbase. The numbers above the branches represent bootstrap support (1000 replications). The analysis was performed in MEGA v4.0 [36]. The *col-12* gene has a *cis*-spliced leader sequence whereas *col-13* has a *trans*-spliced leader. The *cis*-spliced leader sequence is well conserved in one homolog in both *C. remanei* and *C. briggsae*. The near-complete sequence identity between paralogs within genomes is evidence of frequent gene conversion of the coding sequence since the duplication of these genes prior to the divergence of *C. elegans* and the *C. remanei*/*C. briggsae* clade. *Celeg*, *Caenorhabditis elegans*; *Cbrig*, *C. briggsae*; *Crem*, *C. remanei*.

A handful of studies in the pre-genomic era had already hypothesized the occurrence of possible gene conversion events among recently identified *C. elegans* paralogs with extremely high levels of sequence identity. Russnak and Candido [30] hypothesized the formation of the *C. elegans* heat-shock genes *hsp16-1* and *hsp16-48* via an ancestral duplication and inversion event yielding a head-to-head transcriptional orientation. A subsequent round of duplication of this ancestral 1.9 kb module yielded a second *hsp16-1/hsp16-48* cluster 416 bp apart from the ancestral module. The authors favored gene conversion (over an evolutionary recent duplication event) as an explanation to account for the 100% sequence homology between the two *hsp* modules based on the observation that regions flanking the modules were completely diverged in sequence. Actin paralogs *act-1* and *act-3* were also proposed to have undergone concerted evolution via gene conversion and, interestingly, the authors proposed that the conversion events extended to intronic regions in addition to the exonic ones [31]. Another study of two tandem collagen paralogs (*col-12* and *col-13*) inferred an older duplication event leading to their formation based, once again, on conflicting patterns of sequence similarity at the spatial level, extremely low levels of sequence similarity in the flanking regions and 99.5% sequence identity in the open reading frame (ORF) regions including an intron [32]. The identification of *col-12* and *col-13* orthologs in both *C. briggsae* and *C. remanei* and their patterns of sequence identity confirm this conjecture (Figure 1), wherein the two paralogs within each species appear most closely related [33]. 

Several important patterns for the gene conversion process in *C. elegans* paralogs can be gleaned from these initial studies, despite the limited sample sizes. First, gene conversion was concluded to be responsible for the high sequence similarity between paralogous ORFs rather than a recent duplication event because of the low levels or absence of sequence similarity in the flanking regions. Gene conversion could certainly contribute to this observed pattern in paralogous sequences. However, we caution that the possibility of a very recent duplication event should not be ruled out completely without additional evidence. For instance, it is well-documented that gene duplication in *C. elegans* is often incomplete and fails to encompass the entire ORF and/or upstream and downstream flanking regions [34,35]. Extremely high or complete sequence identity between paralogs in their coding regions with diminished sequence homology in flanking regions is concordant with a scenario wherein the gene duplication event was restricted to the ORFs (or an incomplete segment of the ORF) and failed to encompass ancestral flanking region sequences. Second, assuming each of these three studies truly represent interparalogous gene conversion events, it then appears that gene conversion can occur when paralogs are in direct or opposing (head-to-head or tail-to-tail) transcriptional orientation. However, whether *C. elegans* paralogs with the same or opposing transcriptional orientation have equals rates of gene conversion remains to be determined. Third, two of these initial studies reported the extension of gene conversion tracts to introns [31,32]. Fourth, the first estimate of the length of gene conversion tracts in *C. elegans* (>191 bp) was well within the range estimated for other species [25], although with the caveat that gene conversion is characterized by exhibiting a fairly wide range in the length of conversion tracts (several base pairs to >12,000 bp in yeast) and with a high degree of locus-specificity [24]. 

The first comprehensive, genome-wide study of interparalog gene conversion events in nematodes was conducted in 1999 [37]. This landmark study comprised 7829 putative gene duplicates pairs with sequence identity between paralogs ranging from 35–99%. The analysis used the gene conversion detection software GENECONV which tests whether tracts of complete sequence identity between paralogs are larger than expected relative to sequence divergence of DNA sequences flanking these identical segments [38]. Paralogs can display several regions of complete sequence identity either due to (i) multiple gene conversion events, or (ii) in instances where a lengthier conversion tract has incurred mutations resulting in smaller segments of complete sequence identity. Under the latter scenario, each segment of complete sequence identity is considered a gene conversion event. The relatively high sequence identity among these paralogs suggests that these gene duplicates are evolutionarily young and/or have been subjected to sequence homogenization by gene conversion. 526 gene conversion events were detected across the 7829 duplicate pairs surveyed. If locus-bias effects were absent, this would translate into gene conversion affecting 6.7% of the surveyed duplicate pairs. However, only 143 pairs (2% of the sampled duplicates) were affected, with an average of 3.75 conversion events per gene pair. This was the first indication that some duplicate pairs in the *C. elegans* genome are more prone to gene conversion than others. Other relevant findings of the Semple and Wolfe study and more recent studies of interlocus gene conversion in *Caenorhabditis* are discussed in subsequent sections of this review.

## 3. Rate of Gene Conversion in *Caenorhabditis* Paralogs

The rate of gene conversion is an important parameter that determines multiple facets of the evolution of a multigene family. For instance, if the rate of gene conversion is high, significant selective pressure is required for the adaptive diversification of paralogs [6]. Interlocus gene conversion also contributes to allelic variation. Furthermore, gene conversion events with a pseudogene locus as the donor sequence can conceivably impose a significant mutational load on the remaining functional members of the multigene family.

No direct experimental measures of the spontaneous rate of interlocus gene conversion currently exist for *C. elegans*. In experimental populations of *fog-2* mutants, gene conversion where the donor was an upstream paralog of *fog-2* of unknown function was found to be responsible for the reversion of null mutants to wild-type [39]. In the same set of experiments, an exact reversion of the mutation was not detected, suggesting that the rate of gene conversion is at least as high as the base substitution rate. An early study estimated the intralocus/allelic gene conversion at the 38 kb long *unc-22* locus, a gene involved in worm muscle structure and function on Chromosome IV. Intralocus gene conversion events occurred at a frequency of 10^−5^ per locus per generation [40], at a scale similar to the rosy locus in *Drosophila* [41], although it should be cautioned that single-locus estimates of the conversion rate can be a poor proxy for predicting the genome-wide rate given the wide variation (0.8–30% in yeast) in the conversion frequency of different loci [4,42,43]. Moreover, studies in yeast have found that the intralocus/allelic rate of gene conversion exceeds that of interlocus conversion. For example, *his3* paralogs in yeast exhibit an interlocus gene conversion rate of 0.5% relative to an intralocus conversion rate of 1.5% [17].

Reliable measures of the rate of spontaneous interlocus gene conversion events and their genomic characteristics are best answered by analyzing long-term mutation accumulation lines maintained under relaxed selective conditions with periodic assays of a subset of paralogs at different generations.

## 4. Genomic Characteristics of Interlocus Gene Conversion Events in *Caenorhabditis*

### 4.1. Range of Conversion Tracts

A handful of studies in *Caenorhabditis* have provided measures of gene conversion tract lengths, although derived from exceedingly different types of experiments (Table 1). These include empirical estimates from transgenic DNA manipulation [44], global analysis of *C. elegans* paralogs in the sequenced N2 genome with sequence identities ranging from 35–99% [37], bioinformatic analysis of the HSP16 and *zim/him-8* multigene families in sequenced *Caenorhabditis* genomes [45,46] and analysis of paralogs *fog-2/ftr-1* of the FTR gene family in both laboratory experimental evolution lines and natural isolates of *C. elegans* [39,47].

The mean and range of the gene conversion tracts in *Caenorhabditis* paralogs are remarkably consistent across these disparate data sets and different species within the genus, even though gene conversion is known to be site- and context-dependent in other species such as yeast [24,25]. It appears that while individual gene conversion tracts can span a few nucleotides to several thousand, the distribution of surviving gene conversion tracts tend to aggregate towards 50–200 nucleotides. 

**Table 1:** Mean and range of gene conversion tracts in *Canenorhabditis* paralogs.

Paralogs Sampled	Species	Mean (bp)	Range (bp)	Reference
*Tc1* excision repair of the *unc-22* locus	*C. elegans*	>191	>191	[44]
7,829 duplicate pairs	*C. elegans*	117	12–2,958	[37]
*fog-2/ftr-1*	*C. elegans*	58	39–75	[39]
		133^*^	121–145	[39]
		98^**^	93-102	[47]
HSP16 gene family	*C. elegans*	60	60	[45]
	*C. briggsae*	416	44–1113	
*zim/him-8* gene family	*C. elegans*	206223	70–524	[46]
	*C. briggsae*	112	29–274	
	*C. remanei*	223	136–361	

* Based on spontaneous gene conversion in *C. elegans* experimental evolution lines.

** Based on polymorphism in *C. elegans* natural isolates.

However, it should be stressed that the length of the observed gene conversion tracts detected from DNA sequence data may not reflect their original span immediately following the occurrence of the gene conversion event. When a gene conversion event creates a new allele, this novel allele will recombine with other existing alleles in the population, thereby eroding the original tract. Hence, base substitutions originating in a conversion event may get fixed in very limited tracts, even as single nucleotides. The longer conversion tracts seen in the genome may have culminated from the fixation of multiple, short conversion tracts in regions where conversion is not opposed by divergent selection or “terminator mutations” as described by Walsh [5]. 

### 4.2. Gene Conversion in Introns

Gene conversion events between paralogs are usually reported for exonic regions of genes. Indeed, intron sequences diverge faster over evolutionary time in comparison to other parts of a gene and may therefore impede gene conversion events by not meeting the threshold for high sequence homology between the template and donor sequences. Results gleaned from studies of the gene conversion process in the yeast *Saccharomyces cerevisiae* are likely to have the least applicability with respect to the extent that introns are included in gene conversion events, given that the *S. cerevisiae* genome is especially depauperate in introns (only 4% of coding genes) [48].

There is some evidence that gene conversion events in *C. elegans* often encompass intronic sequences. Two of the initial studies postulating gene conversion events in *C. elegans* paralogs reported the extension of gene conversion tracts to introns [31,32]. More recent studies provide additional evidence that gene conversion seems to extend to introns in *Caenorhabditis* genomes. Intron position, phase and sequence were conserved for cytoplasmically localizing *hsp70* paralogs in both *C. elegans* and *C. briggsae* [49]. Likewise, gene conversion between paralogs of the *zim/him-8* tandem cluster also encompasses one intron [46]. Semple and Wolfe [37] were able to identify an extension of the gene conversion event to intron(s) in 15% of all detectable gene conversion events, while acknowledging that their method was based on analysis of coding regions and hence biased against the detection of gene conversion in intronic regions. The inclusion of intronic sequences in gene conversion tracts may be facilitated by the presence of relatively compact introns in the *C. elegans* genome and other Caenorhabditids, the modal and median intron sizes in *C. elegans* being a mere 47 and 65 bp, respectively [50].

### 4.3. Influence of Genomic Distance and Transcriptional Orientation

The genomic proximity of paralogous genes is often thought to facilitate gene conversion [14,37]. However, a large fraction of duplicates in diverse genomes often occur in genomic proximity as adjacent loci with the same transcriptional orientation. A recent study of ectopic gene conversion events in the human genome found no evidence for greater conversion frequencies between closely-spaced paralogs when the distribution of gene-family members is controlled for [51]. Genomic proximity or distance can be measured at two levels. First, duplicate pairs can be classified on the basis of the chromosomal location of the two paralogs (interchromosomal *versus* intrachromosomal, signifying residence of the two paralogs on different chromosomes or the same chromosome, respectively). Second, for paralogs existing on the same chromosome, genomic proximity can be further measured on the basis of the physical distance (in base pairs or number of loci) separating them. In our discussion, we use the term ‘tandem’ to describe two paralogs with no intervening loci and in the same transcriptional orientation. In the literature, ‘tandem’ has been interchangeably used to describe adjacent paralogous loci irrespective of transcriptional orientation and this has garnered some confusion. We further distinguish ‘adjacent inverted’ paralogs to signify gene duplicates with no intervening loci but in opposite transcriptional orientation. 

The majority of studies providing evidence of gene conversion in *Caenorhabditis* paralogs have tended to focus on a single duplicate pair or a small multigene family, often existing as adjacent loci on a chromosome with instances of both inverted (head-to-head or tail-to-tail) and direct (same) orientation. Some of these studies include two *C. elegans hsp16-48* modules existing as an inverted repeat separated by 416 unique bp [30], tandem collagen paralogs *col-12* and *col-13* separated by 1.8 kb [32], adjacent inverted heat-shock genes *hsp70-7* and *hsh70-8* [49], and tandem FTR-family paralogs *fog-2* and *ftr-1* separated by 763 unique bp [39,47]. So it is apparent that gene conversion events can occur irrespective of the transcriptional orientation of the paralogs in question, when in genomic proximity as adjacent loci in the genome. However, the limited sample sizes of these studies preclude the determination of the relative frequencies of gene conversion for paralogs exhibiting the same *versus* opposite transcriptional orientation.

Semple and Wolfe’s [37] global analysis of 7829 *C. elegans* duplicate pairs with moderate to high sequence identity found a significant negative correlation between gene conversion frequency and distance (ranging from 1–8 kb) for converted paralogs residing on the same chromosome. They further suggest that gene conversion events are more likely between paralogs bearing the same transcriptional orientation. In their data set of intrachromosomal duplicate pairs, 80% of the neighboring duplicates (paralogs separated by <5 genes) and 84% of the adjacent duplicates (no genes separating the paralogs) exhibited conservation of transcriptional orientation between the paralogs. However, it is not clear from this analysis if the enhanced frequency of gene conversion events for these loci can be attributed independently to an effect of orientation without the confounding effects of genomic proximity. Their study also provided the first robust estimates of the relative frequencies of *intra*- *versus* *interchromosomal* gene conversion events in *C. elegans* paralogs. The rate of intrachromosomal gene conversion (referred to as “*cis*” duplications) in *C. elegans* paralogs was determined to be 3.7-fold higher than interchromosomal (or “*trans*” duplications) gene conversion, and in a ratio similar to those observed in yeast (~3.0) [14], mice (~2.9) [52] and *Drosophila* (~6.0) [53].

### 4.4. Influence of Family Size and Sequence Homology

It would appear intuitive that the greater the population of possible homologous donors for conversion events at a particular locus, the greater the probability of it experiencing recombination events such as gene conversion. Indeed, studies in yeast have reported that the frequency of gene conversion in a particular yeast gene is directly proportional to the number of identical donor sequences present [54]. In contrast, a prominent (and unexpected) conclusion stemming from Semple and Wolfe’s [37] analysis of *C. elegans* paralogs was the existence of a significant negative correlation between gene conversion frequency and multigene family size. The authors speculated that an increase in the number of potential donor sequences might result in a concomitant increase in the incidence of gene conversion and a lowered rate of detection. One can foresee that a sufficiently high frequency of gene conversion would likely result in multiple gene conversion hits at the recipient locus. If these gene conversion tracts are overlapping or closely-spaced along the length of the recipient locus, it will likely appear as a single lengthy conversion tract originating from a single gene conversion event, and lead to an underestimate of the actual gene conversion frequency and an overestimate of the length of the conversion tract.

The degree of sequence identity and the length of homology between the donor and recipient sequences are major determinants of the frequency of gene conversion. Semple and Wolfe’s study [37] confirms this pattern for *C. elegans* paralogs, wherein they find a significant negative correlation between gene conversion and DNA sequence divergence. We currently lack a robust estimate for the minimum sequence identity required to facilitate gene conversion between two homologous sequences in *Caenorhabditis* genomes. However, the average sequence identity between paralogs may not be the appropriate parameter. Two paralogous sequences can have moderate levels of overall sequence divergence but the variable sites may not be uniformly distributed along the length of the genes. Gene conversion can still occur between such genes in interspersed regions of high sequence identity. As an example, we consider gene conversion events detected in *C. elegans* tandem paralogs *fog-2/ftr-1* with an estimated overall synonymous sequence divergence (*K*_S_) of 28% using the maximum-likelihood procedure in the PAML package [55]. In a gene conversion event of *fog-2* via *ftr-1*, the conversion tract extended downstream into a 72 bp region of 100% sequence identity as well as an upstream region of 77% sequence identity [39]. 

It therefore appears that for linked sequences such as adjacent paralogous genes, the genomic proximity may facilitate gene conversion despite moderate sequence divergence between the sequences and shorter tracts of complete identity. In contrast, higher thresholds of sequence identity and homology length may be required to facilitate interchromosomal gene conversion events in more complex genomes such as those of multicellular eukaryotes. For example, efficient levels of ectopic gene conversion between unlinked sequences in mice required 2.5 kb of identical sequence and diminished drastically when tract length was reduced to 1.2 kb [56]. 

## 5. Population-Genetic and Phenotypic Effects of Interlocus Gene Conversion in *C. elegans*

**Figure 2:**
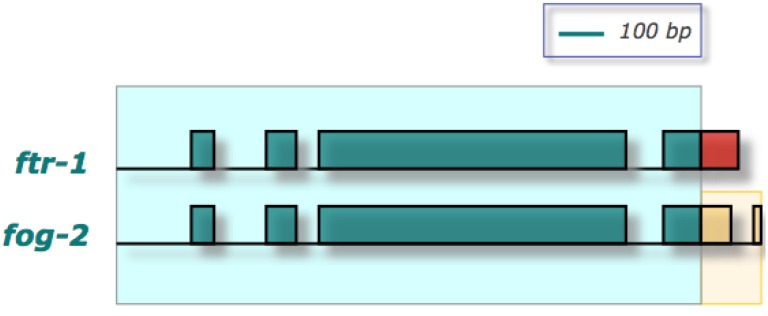
Schematic depicting the regions of homology between the paralogs *fog-2* and *ftr-1* [39,47]. Shaded rectangles denote exons; horizontal lines represent introns and duplicated flanking regions where applicable. The duplicated region is shaded in light blue. Duplicated segments as determined by sequence homology between the two paralogs are also depicted by the correspondence of regions with identical color and pattern. Nonhomologous segments of the two paralogs are depicted in different colors. The novel portion of *fog-2* highlighted in yellow likely confers the novel function of hermaphrodite spermatogenesis.

Despite a plethora of studies that have found evidence of gene conversion in DNA sequences across a diverse set of organisms, the phenotypic consequences of such gene conversion events are largely obscure. In experimental populations of *C. elegans*, a gene conversion event led to the repair of a loss-of-function mutation with an extreme phenotypic consequence of sex reversion [39]. *C. elegans* is one of two species in its genus exhibiting an androdioecious mode of reproduction with populations composed largely of self-fertile hermaphrodites and males in low-frequency [57]. *C. elegans* hermaphrodites phenotypically resemble the females of other congeneric, obligately-outcrossing gonochoristic species but have evolved the ability to produce limited amounts of sperm for self-fertilization. The evolution of hermaphroditism in *C. elegans* may have been specifically promoted by the appearance of a new gene, *fog-2*, via a gene duplication of *ftr-1* that enabled spermatogenesis in *C. elegans* hermaphrodites [39,58,59]. *ftr-1*, a gene of unknown function, comprises four exons encoding 314 amino acids (aa). The exon-intron structure of *fog-2*, comprising five exons (327 aa) exhibits both similarities and dissimilarities relative to *ftr-1* (Figure 2). Homology between *ftr-1* and *fog-2* commences ~170 bp upstream of the start codon, completely encompassing the first three exons and introns and partially spanning the terminal exon of *ftr-1* (first 91 of 186 bp). Synonymous sequence divergence between the two paralogs over their region of homology is approximately 28%. The novel terminal region of *fog-2* spans the latter 66 bp of exon 4, the entire intron 4 (45 bp) and entire exon 5 (157 bp) [39]. The creation of *fog-2* likely resulted from a *partial duplication with recruitment* [35] of *ftr-1* with the incorporation of novel sequence into its reading frame at the C-terminus from it new genomic neighborhood, likely conferring the novel function of hermaphrodite spermatogenesis in this species [47,59]. Both paralogs reside in tandem on Chromosome V, separated by 763 bp of unique sequence, with *ftr-1* placed directly upstream of *fog-2* [39]. 

**Figure 3:**
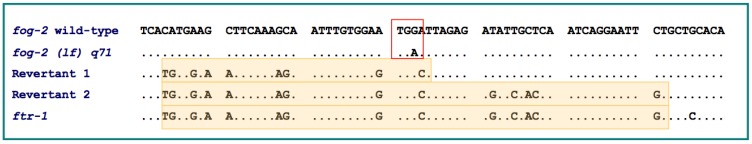
Sequence alignments of in-frame nucleotide positions 200-499 of exon 3 displaying two independent gene conversion events at the *fog-2* locus by *ftr-1* resulting in a phenotypic alteration from obligate outcrossing to hermaphroditism in two *fog-2(lf) q71* mutant lines [39]. The red box displays the nonsense mutation G → A in the *fog-2(lf)q71* allele resulting in a nonfunctional gene relative to the wild-type. Regions highlighted in yellow represent the minimum gene conversion tracts by *ftr-1* in sex-revertants 1 and 2. Dots represent identical nucleotides to the *fog-2* wild-type sequence.

A long-term mutation-accumulation study by us employed replicate lines of *C. elegans* rendered obligately-outcrossing (male-female) by a loss-of-function (*lf*) mutation in the *fog-2* coding region due to the presence of a premature stop codon. During the course of this experiment, several of these male-female lines reverted spontaneously to hermaphroditism during the population bottleneck phase. Hermaphroditic revertants also originated and reached fixation in a handful of lines during a subsequent fitness-recovery phase of the experiment. Two of the sequenced revertants to hermaphroditism were established to have resulted from a gene conversion event whereby a short segment of a paralogous gene, *ftr-1*, recombined with the *fog-2 (lf)* mutant allele, replacing the premature stop codon with a tryptophan codon, thereby restoring *fog-2* function and leading to the subsequent appearance of functional selfing hermaphrodites (Figure 3) [39]. This type of repair of a deleterious mutation by gene conversion has been suggested to be important in the evolutionary conservation of the human Y chromosome, where rampant gene conversion between paralogs may have stalled Y chromosome degeneration [60,61] and deleterious mutation accumulation in plastid genomes [62]. The overall nucleotide sequence identity between *ftr-1* and *fog-2* over their homologous coding region is 82%; however, the converted region in *fog-2* in the experimental populations is downstream of a stretch of 70 bp of complete identity that probably facilitated these events. Moreover, the sequence tract that was converted in the experimental lines had an overall nucleotide sequence identity of 77% between *fog-2* and *ftr-1.* Considering how easily these spontaneous gene conversions were obtained in experimental populations, the fact that this region has remained impervious to gene conversion over evolutionary time suggests functional divergence between the two genes in this region.

**Figure 4:**
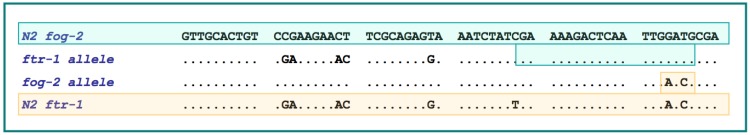
Evidence of gene conversion in both *fog-2* and *ftr-1* alleles in natural isolates of *C. elegans* [47]. The top and bottom sequences (highlighted in green and yellow throughout, respectively) represent base positions 470-589 of the *fog-2* and *ftr-1* alleles of the N2 laboratory strain. Dots represent nucleotides identical to the N2 *fog-2* sequence. The second sequence labeled ‘*ftr-1* allele’ represents a gene conversion event in *ftr-1* by a *fog-2* donor sequence. The minimum gene conversion tract length of 9 bp in this converted *ftr-1* allele is highlighted in green to display its sequence identity to the N2 *fog-2* allele. The region surrounding this converted tract has 100% sequence identity between N2 *fog-2* and *ftr-1* alleles over a 102 bp stretch, which would represent the maximum length of the gene conversion tract. This converted *ftr-1* allele was detected in 20 of the 40 natural isolates surveyed. The third sequence labeled ‘*fog-2* allele’ represents a gene conversion event in *fog-2* by an *ftr-1* donor sequence possessed by three of 40 natural isolates. The minimum gene conversion tract length of 3 bp in this converted *fog-2* allele is highlighted in yellow to display its sequence identity to the N2 *ftr-1* allele. The region surrounding the converted nucleotides is identical between *fog-2* and *ftr-1* over a span of 93 bp, representing the maximum possible length of the gene conversion tract.

Interlocus gene conversion events serve to increase allelic diversity at a locus and may result in shared polymorphisms between loci [63,64,65]. In a follow up study, *Rane et al.* [47] further analyzed DNA sequence variation in *fog-2* and *ftr-1* within 40 isolates of *C. elegans* to investigate the population-genetic consequences of gene conversion in natural populations (Figure 4). Gene conversion was found to contribute significantly to DNA sequence diversity at *fog-2* (22%) and *ftr-1* (34%) in these populations. This study found a region of shared polymorphism between the two paralogs that was positioned immediately upstream of a 75 bp gene conversion tract. It appears that this stretch of nucleotides, with 100% identity in the two paralogs, is facilitating gene conversion in both experimental [39] and natural populations of *C. elegans*.

In conclusion, the population-genetic consequences of gene conversion are to increase allelic diversity at paralogous loci. The standing genetic variation at synonymous sites within a species is frequently utilized to estimate the parameter *N*_e_*μ* as a proxy for the species effective population size (*N*_e_). Estimates of effective population size (*N*_e_) for a species calculated from the standing genetic variation at a converted locus will lead to inflated estimates.

## 6. Evolutionary Consequences of Ectopic Gene Conversion for *Caenorhabditis* Multigene Families

Preceding studies have established interlocus gene conversion as an important homogenizing force in the evolution of gene duplicates in *Caenorhabditis* species. Semple and Wolfe [37] found evidence for gene conversion in only 2% of 7,829 duplicate pairs in the *C. elegans* genome, and 85% of these events were restricted to members of multigene families. This may have fostered the notion that gene conversion is a less potent force in the evolution of small gene families, comprising <5 paralogs [34]. However, statistical methods [38,66] currently available for the detection of gene conversion events between paralogs in the absence of an outgroup sequence fail to work under regimes of high sequence identity between the focal paralogs. Most statistical analyses of gene conversion events in *C. elegans* have employed Sawyer’s GENECONV statistical software [37,39,45,47] which uses the distribution of mismatches between DNA sequences to test the null hypothesis of no gene conversion between paralogs. GENECONV performs well in simulation studies but is found to be lacking in power under regimes of frequent gene conversion [67]. Hence, the view that gene conversion in *C. elegans* is relatively infrequent may need to be reevaluated. Analysis of laboratory-based mutation accumulation lines, with the strength of selection reduced to a minimal, ought to yield the most robust estimate of the spontaneous rate of gene conversion between paralogs in *Caenorhabditis* genomes, the length of gene conversion tracts, and the influence, if any, of key genomic characteristics (transcriptional orientation, chromosomal location, extent of sequence homology) on this key parameter. 

We next consider the evolutionary impacts of gene conversion on the functional fate of gene duplicates. Under environmental conditions where increased gene dosage is beneficial, natural selection is expected to favor concerted evolution of paralogs via gene conversion [68,69,70]. On the flip side, if sequence homogenization of paralogous genes via gene conversion is sufficiently frequent, it begs the question as to how duplicates are ever able to establish independent evolutionary trajectories and neofunctionalize. With increasing sequence divergence, gene conversion between paralogs is expected to eventually taper in frequency, enabling the probability of functional divergence [5]. However, we have no estimate for this threshold of sequence divergence between *Caenorhabditis* paralogs that would serve to decrease the frequency of gene conversion to levels that effectively render it evolutionarily impotent. Even more intriguing is the question as to how this threshold of sequence divergence is ever reached under the onslaught of frequent gene conversion. Earlier theoretical work exploring the conundrum of gene duplicate neofunctionalization in the face of gene conversion pressure have suggested that “terminator mutations” such as large insertions/deletions, obstruction of sequence homology via mobile element insertion, or translocation of one paralog to another chromosome via reverse transcription may serve to interrupt sequence homology between paralogs and retard further homogenization of the two copies [5]. More recent theoretical studies have explored the role of diversifying natural selection in the maintenance of paralog sequence diversity under conversion pressure [6]. The patterns of DNA variation in human antigen-coding paralogs RHCE and RHD appear consistent with a model of selection maintaining antigen diversity despite frequent gene conversion, although the strength of selection required to counterbalance homogenization by gene conversion was inferred to be extremely high [6].

We additionally suggest that structural heterogeneity between paralogs is yet another factor that likely plays an important role in restricting complete homogenization of paralogs via gene conversion, thereby promoting neofunctionalization. The mechanisms of gene duplication often fail to respect gene boundaries, resulting in the duplication of gene fractions rather than the complete ORFs of the ancestral copy [34,35]. More than 50% of gene duplicates with synonymous sequence divergence less than 10% in the *C. elegans* genome comprised structurally heterogeneous paralogs, wherein one or both copies had unique exonic regions to the exclusion of their sister copy [34]. If these unique coding regions in one or both paralog(s) encode novel functional domains, neofunctionalization can be promoted and maintained despite ongoing gene conversion in homologous regions of the two paralogs. As a case and point, we revisit the *fog-2/ftr-1* scenario (Figure 2). The putative ancestral copy, *ftr-1*, is of unknown function. *fog-2*, implicated in the origin of hermaphroditism in *C. elegans*, likely originated from a partial duplication of *ftr-1* that prematurely terminated in the terminal exon of *ftr-1*, and then recruited additional unique sequence from its new genomic neighborhood to complete its ORF [39]. Intriguingly, the recruitment of this unique sequence in the 3' end of *fog-2* likely facilitated its neofunctionalization after duplication [47]. Frequent gene conversions of *fog-2* by *ftr-1* in both laboratory and wild *C. elegans* populations fail to diminish or compromise the function of *fog-2* in hermaphrodite spermatogenesis, given that this neofunctionalized sequence tract in *fog-2* bears no homology to *ftr-1* sequence.

## 7. Conclusions

Independence of mutations is an important assumption in much of evolutionary genetic analysis. Gene conversion and other mechanisms of reticulate evolution resulting in shared mutations between loci can adversely affect the results of evolutionary analyses in a number of different ways. First, gene conversions can obscure and mislead conclusions on the phylogenetic relationship between genes. Second, gene conversions can lead to gene duplicates appearing more evolutionarily recent than they really are, leading to erroneous calculations with respect to the dynamics of duplicates genes in genomes. Third, gene conversion appears to occur more frequently between paralogs residing in genomic proximity and can thereby skew our understanding of gene movement around the genome. Fourth, gene conversion can result in inferences of selection when there are none [71]. Lastly, gene conversion serves to increase allelic diversity leading to inflated estimates of the effective population size (*N*_e_) in instances where base substitution parameters are used to infer *N*_e_.

Semple and Wolfe’s study found evidence of gene conversion in only 2% of gene duplicates in the *C. elegans* genome [37]. GENECONV software [38], often used to statistically detect the presence of gene conversion between paralogs, may underestimate gene conversion frequencies for sequences with low divergence, representing a pool of duplicates most likely to undergo conversion. Hence, the view that gene conversion is infrequent in *C. elegans* may need to be revaluated. Due to low levels of DNA sequence variation in natural populations of *C. elegans*, shared polymorphisms between paralogs (a key signature of gene conversion) in this species may be more challenging to detect compared to other species such as *Drosophila*. High-throughput sequencing of *C. elegans* mutation accumulation lines will eventually yield data on the spontaneous gene conversion rate whereas a combination of methods will be needed to evaluate the role of gene conversion in the evolution of *Caenorhabditis* genomes. These will undoubtedly take advantage of various *Caenorhabditis* genome sequencing projects currently in progress by analyzing relationships of paralogs whose synteny context has been conserved since speciation.
